# Incidental esophageal submucosal tumor detection by chest radiography: A case report

**DOI:** 10.3892/etm.2014.2008

**Published:** 2014-10-08

**Authors:** SOSUKE TADANO, TADASHI KONDO, MUNEAKI WATANABE, NORIO TAKAYASHIKI, TOSHIHIRO SHIOZAWA, GEN OHARA, KASTUNORI KAGOHASHI, KOICHI KURISHIMA, HIROAKI SATOH

**Affiliations:** 1 Division of Surgery, Mito Medical Center, University of Tsukuba, Mito, Ibaraki 310-0015, Japan; 2 Division of Pathology, Mito Medical Center, University of Tsukuba, Mito, Ibaraki 310-0015, Japan; 3 Division of Respiratory Medicine, Mito Medical Center, University of Tsukuba, Mito, Ibaraki 310-0015, Japan

**Keywords:** esophageal submucosal tumor, esophageal leiomyoma, mass-screening, chest radiograph

## Abstract

Esophageal submucosal tumors are occasionally detected incidentally during a gastrointestinal survey. In the present study, a case of esophageal leiomyoma is reported, which was incidentally detected by chest radiography during an annual survey of mass-screening for lung cancer. The patient underwent a laparoscopic lower esophagectomy, a proximal gastrectomy and a gastric tube reconstruction. Macroscopic examination revealed a 50×40×28-mm mass, while microscopic examination identified submucosal smooth muscle tissue without mitotic activity or necrosis. The tumor was diagnosed as an esophageal leiomyoma. The patient was asymptomatic during the three-month follow-up period. However, when a mass lesion adjacent to the gastrointestinal tract is detected during chest radiography, the possibility of a rare disease should be considered. Therefore, further investigation with upper gastrointestinal radiography and gastroendoscopy should be performed.

## Introduction

Leiomyomas are the most common esophageal mesenchymal neoplasms, even though their occurrence is rare. Patients with esophageal leiomyomas are usually asymptomatic. The most common symptoms, when present, include epigastric discomfort, dysphagia, regurgitation, gastrointestinal bleed, diarrhea and weight loss. Esophageal leiomyomas are occasionally detected incidentally during the examination of other gastrointestinal diseases, of which the majority are identified during endoscopic examination or upper gastrointestinal radiography (1–4). Considering the tumor size and position, patient’s symptoms, general condition and comorbidities, surgical treatment should be determined. In the present study, the case of an esophageal leiomyoma is reported, which was incidentally detected on a plain chest radiograph performed during an annual survey of mass-screening for lung cancer.

## Case report

**Figure 1:**
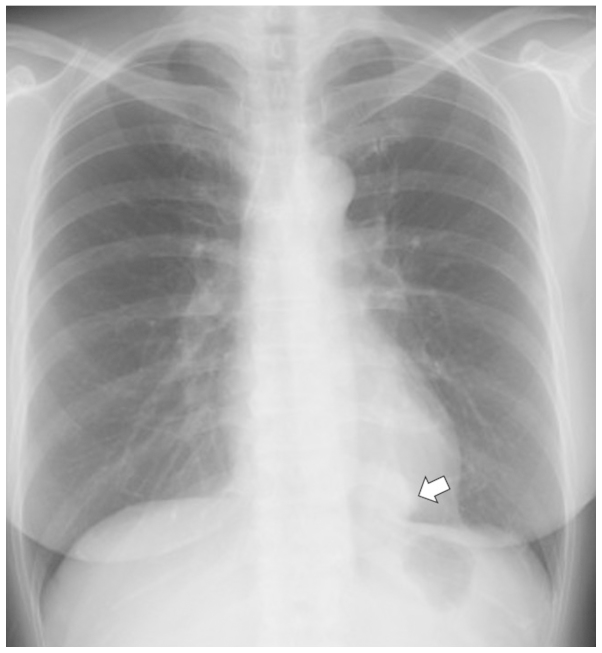
Mass shadow (arrow) detected on a chest radiograph during a mass-screening program.

**Figure 2:**
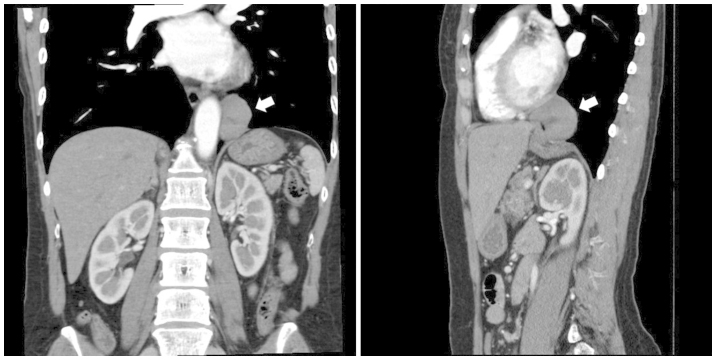
Computed tomography scan revealed a mass (arrow) at the distal esophagus.

**Figure 3:**
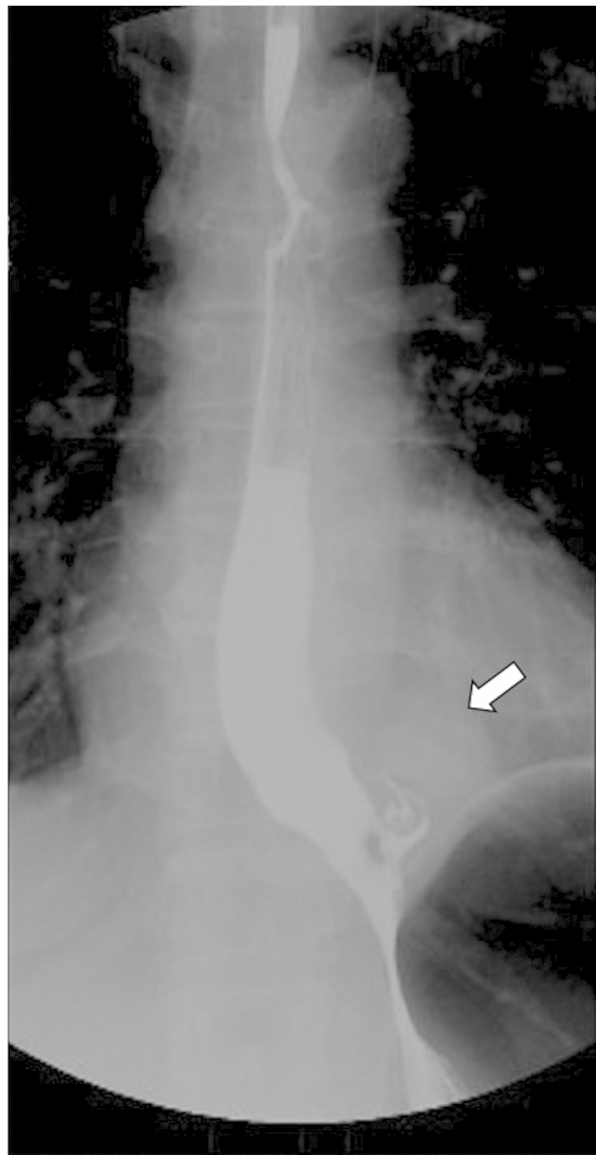
Upper gastrointestinal tract radiography revealed an esophageal submucosal tumor (arrow) at the distal esophagus.

**Figure 4:**
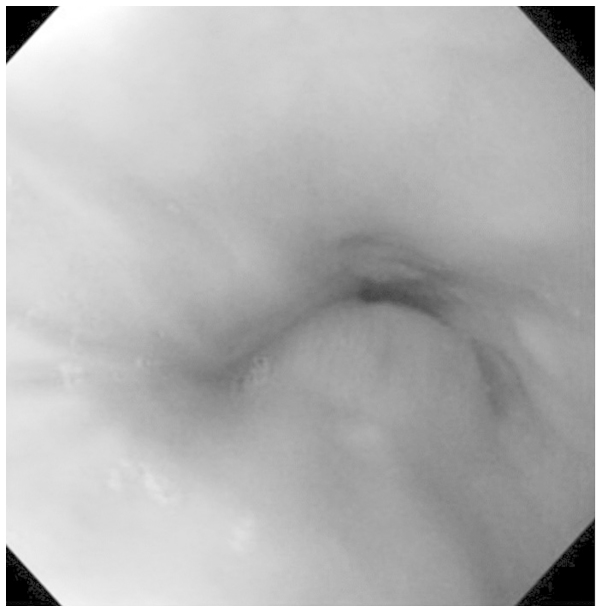
Upper gastrointestinal endoscopy revealed a submucosal tumor with a normal mucosa.

**Figure 5:**
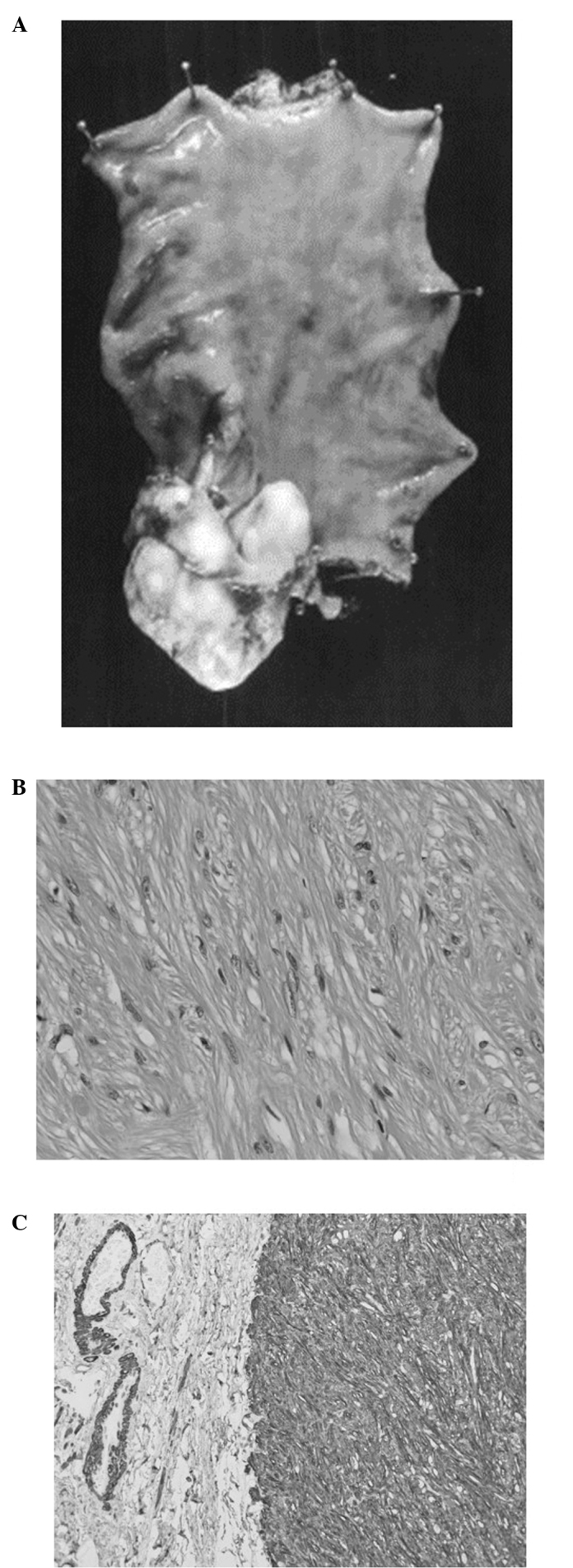
Images of the resected esophageal submucosal tumor (SMT). (A) Gross inspection of the resected esophageal SMT. (B) Histopathology of the resected tumor (×200; hematoxylin-eosin staining). (C) Immunostaining with α-smooth muscle actin (×100; hematoxylin-eosin staining).

A 55-year-old female was referred to the Mito Medical Center (Mito, Japan) following the detection of a nodule on a chest radiograph performed during an annual survey of mass-screening (Fig. 1). The mass-screening was performed one month prior to this study. The patient did not present any symptoms, such as dysphagia or epigastric pain, had never smoked and had no previous medical history. Physical examination was unremarkable and the results of standard laboratory tests were normal. A chest and abdominal computed tomography (CT) scan (Aquilion 64; Toshiba, Tokyo, Japan) detected a nodule measuring 5 cm in diameter at the distal esophagus, without invading the cardia of the stomach (Fig. 2). An upper gastrointestinal tract radiograph revealed an esophageal submucosal tumor (SMT) at the distal esophagus (Fig. 3), and upper gastrointestinal endoscopy (GIF-H290; Olympus Corporation, Tokyo, Japan) confirmed the diagnosis of an esophageal SMT with a normal mucosa (Fig. 4). Histopathological examination of the tumor biopsy revealed spindle cell proliferation; however, mitotic activity or cellular anaplasia were not detected. Immunohistochemical analysis revealed diffuse and strong positive staining for α-smooth muscle actin (Dako, Tokyo, Japan). No enlargement of the adjacent lymph nodes or evidence of distant metastasis were identified on the chest and abdominal CT scans. The patient was subjected to a laparoscopic lower esophagectomy, a proximal gastrectomy and a gastric tube reconstruction. Macroscopic examination revealed a 50×40×28-mm mass, while microscopic examination identified submucosal smooth muscle tissue without mitotic activity or necrosis (Fig. 5). The patient was asymptomatic during the three-month follow-up period. Written informed patient consent was obtained from the patient.

## Discussion

Esophageal SMTs are occasionally detected incidentally during the examination of other gastrointestinal diseases (1–4). In these cases, the majority have been identified by upper gastrointestinal tract radiography (2) or endoscopy (1,3,4). Esophageal leiomyomas may appear as a posterior mediastinal mass on chest radiographs (5–7); thus, are identified as an incidental radiographic finding (8,9). In the present study, the right lower lung nodule was initially suspected to be a primary lung cancer tumor, an arteriovenous malformation or a gastrointestinal stromal tumor due to the nodule shape and position.

Common symptoms of an esophageal leiomyoma include dysphagia, epigastric pain and retrosternal pain or burning. However, the leiomyoma grows slowly and half of the patients do not present any symptoms unless the tumor is intramural (5,7). Bleeding is common in patients with gastric leiomyomas, whereas esophageal leiomyomas do not ulcerate and rarely bleed (7,10). The patient in the current study did not show any symptoms, such as dysphagia or epigastric pain.

The diameter of esophageal leiomyomas has been reported to be <5 cm in 49% of cases, 5–9 cm in 33.7% of cases and >10 cm in 17.3% of cases (11). Several studies have recommended the observation of asymptomatic patients with lesions of <5 cm at the tumor’s largest diameter, as well as when the preoperative analysis excluded malignancy (11,12). However, other studies have reported that malignancy is unable to be completely excluded prior to surgery (5,10). In the present study, the patient did not present any symptoms or evidence of malignancy in the specimens biopsied, although sections of the tumor were >5 cm in diameter. Therefore, a surgical resection of the tumor was performed.

Community-based lung cancer screening using chest radiography is well-established in Japan (13–15), allowing residents aged ≥40 years to undergo an annual chest radiography. This screening program has been supported by the Japanese national government under the Health and Medical Services Law for the Aged since 1987 (16). Participants who are suspected of having lung cancer following a chest X-ray or sputum cytology are offered further examinations to confirm the diagnosis during a secondary evaluation. However, a number of diseases other than primary lung tumors have been diagnosed through the mass-screening program, most commonly benign lung, metastatic lung and mediastinal tumors (17–19).

To the best of our knowledge, the current study presents the first case of an esophageal leiomyoma detected on a chest radiograph during a mass-screening program. In conclusion, a mass lesion adjacent to the gastrointestinal tract that is detected on a chest radiograph may potentially indicate a rare disease. Therefore, further investigation with upper gastrointestinal radiography and gastroendoscopy should be performed.
